# The Effect of Sustained Compression on Oxygen Metabolic Transport in the Intervertebral Disc Decreases with Degenerative Changes

**DOI:** 10.1371/journal.pcbi.1002112

**Published:** 2011-08-04

**Authors:** Andrea Malandrino, Jérôme Noailly, Damien Lacroix

**Affiliations:** Institute for Bioengineering of Catalonia, Barcelona, Spain; Medical College of Wisconsin, United States of America

## Abstract

Intervertebral disc metabolic transport is essential to the functional spine and provides the cells with the nutrients necessary to tissue maintenance. Disc degenerative changes alter the tissue mechanics, but interactions between mechanical loading and disc transport are still an open issue. A poromechanical finite element model of the human disc was coupled with oxygen and lactate transport models. Deformations and fluid flow were linked to transport predictions by including strain-dependent diffusion and advection. The two solute transport models were also coupled to account for cell metabolism. With this approach, the relevance of metabolic and mechano-transport couplings were assessed in the healthy disc under loading-recovery daily compression. Disc height, cell density and material degenerative changes were parametrically simulated to study their influence on the calculated solute concentrations. The effects of load frequency and amplitude were also studied in the healthy disc by considering short periods of cyclic compression. Results indicate that external loads influence the oxygen and lactate regional distributions within the disc when large volume changes modify diffusion distances and diffusivities, especially when healthy disc properties are simulated. Advection was negligible under both sustained and cyclic compression. Simulating degeneration, mechanical changes inhibited the mechanical effect on transport while disc height, fluid content, nucleus pressure and overall cell density reductions affected significantly transport predictions. For the healthy disc, nutrient concentration patterns depended mostly on the time of sustained compression and recovery. The relevant effect of cell density on the metabolic transport indicates the disturbance of cell number as a possible onset for disc degeneration via alteration of the metabolic balance. Results also suggest that healthy disc properties have a positive effect of loading on metabolic transport. Such relation, relevant to the maintenance of the tissue functional composition, would therefore link disc function with disc nutrition.

## Author Summary

Low back pain is a very common pathology in industrialized countries, often due to bad posture. It is also highly related to intervertebral disc aging. Aging of the disc is a normal process characterized by series of changes in its structure and function. The events that convert normal aging into degenerative disease is still not clear. Complications such as limited nutrition and possible reduction of disc cells with age make the issue intricate and multi-factorial. Using a numerical model that includes both nutritional and mechanical components, we found two different situations when looking at the effect of external loadings on two important cell solutes related with disc metabolism: oxygen and lactate. The effect of mechanical loading was greater when compressing a healthy disc than a degenerated one and promoted fluctuations of solutes concentrations. Also, changes in cell density seem fundamental in the process of disc degeneration and its causality with other degenerative changes should be further investigated. The importance of both mechanical and cellular patterns to maintain a healthy condition provides new insights to the field of disc regenerative medicine.

## Introduction

Degenerative changes of the intervertebral discs (IVDs) occur either in a pathological manner or as a consequence of aging, and seriously compromise the tissue capability to sustain the stressful loads transmitted throughout the spine [1], [2]. The IVD is the largest avascular tissue in our body and is maintained by a relatively small number of cells, which further decreases with age [3]. Therefore, disc degenerative changes (DDCs), are strongly suspected to be linked with a failure of nutrient transport from the peripheral blood vessels to the IVD cells [4]. Nutrient transport within the disc depends on the tissue composition and morphology, and is also coupled with the response to mechanical loads. While it is reported [5]–[7] that sustained mechanical stresses affect transport of solutes and that the nutrient pathway disturbance acts concomitantly to degenerative phenomena [4], [8], [9], it has not been clearly investigated whether and how DDCs could affect solute transport.

Anaerobic glycolysis has been recognized as the main source of energy for disc cells. Experimental work led to empirical equations modeling the interactions between the main two nutrients, i.e. oxygen and glucose and the relevant metabolic waste product, lactate [10]. For small neutral solutes such as oxygen, lactate and glucose, diffusion has been underlined as the main transport mechanism [11], [12]. The effect of static compression on diffusion of Gadoteridol (a small solute) in the IVD has also been recently studied *in vivo*, suggesting a load-dependence process for the diffusion of the solute [5].

Due to the difficulty of measuring *in vivo* the solute distributions within the IVD [13], finite element (FE) modeling of transport processes is often used to complement experiments and bring further insights in disc nutrition and degeneration issues. Such computational studies have shown the importance of biochemical couplings in the disc glycolytic metabolism [14], and that of endplate obstruction to nutrients and waste products due to calcification and sclerosis [15]. Fluid velocity (i.e. advective transport) was suggested to have a negligible role in enhancing small solute transport [16]. Nevertheless, by coupling metabolic reactions together with a multiphasic mechanical model, different effects on solute concentrations (oxygen and lactate) were found induced by static and dynamic compressions [17], suggesting a potential role of fluid advection enhancement. Using a similar theoretical framework, Magnier and coworkers [18] studied the disc solute transport and the effect of mechanical coupling under free swelling. The simulated transport process depended on porosity, cell density and endplate diffusion area, but was independent of disc stiffness. Nevertheless, under sustained loads and significant tissue deformation, solute transport is expected to depend on disc stiffness.

Most of the abovementioned numerical studies did not consider any local strain-dependent diffusivity [16], [19], [20] and advective effect was not clearly studied [15], [19], [21]. Moreover, the few models that included both load-dependent advection and diffusion were coupled to bi-dimensional simplified geometries [17], [18], though considering 3D geometries may substantially change the transport predictions [19]. Since none of the abovementioned models fully coupled the diffusive, convective, and metabolic 3D transport equations with large mechanical strains, the metabolic solute transport interactions with multiple DDCs, i.e. loss of fluid proteglycan content, solid matrix stiffening, cell death, geometrical changes, etc. could not be properly explored. As such, a model including biphasic mechanics, advection-diffusion-reaction (ADR), and 3D geometry is needed to better understand IVD nutrient transport in healthy and pathological situations.

We aimed at contributing to the intricate mechanics-transport connections involved in the pathophysiology of the human IVD by using a coupled mechanics-ADR approach in a 3D FE model. First, we hypothesized that mechano-transport couplings are essential to predict the solute distributions in the healthy and degenerated discs. Second, we parametrically assessed which DDC would mostly affect the oxygen and lactate transport, and in which manner.

## Results

**Figure 1:**
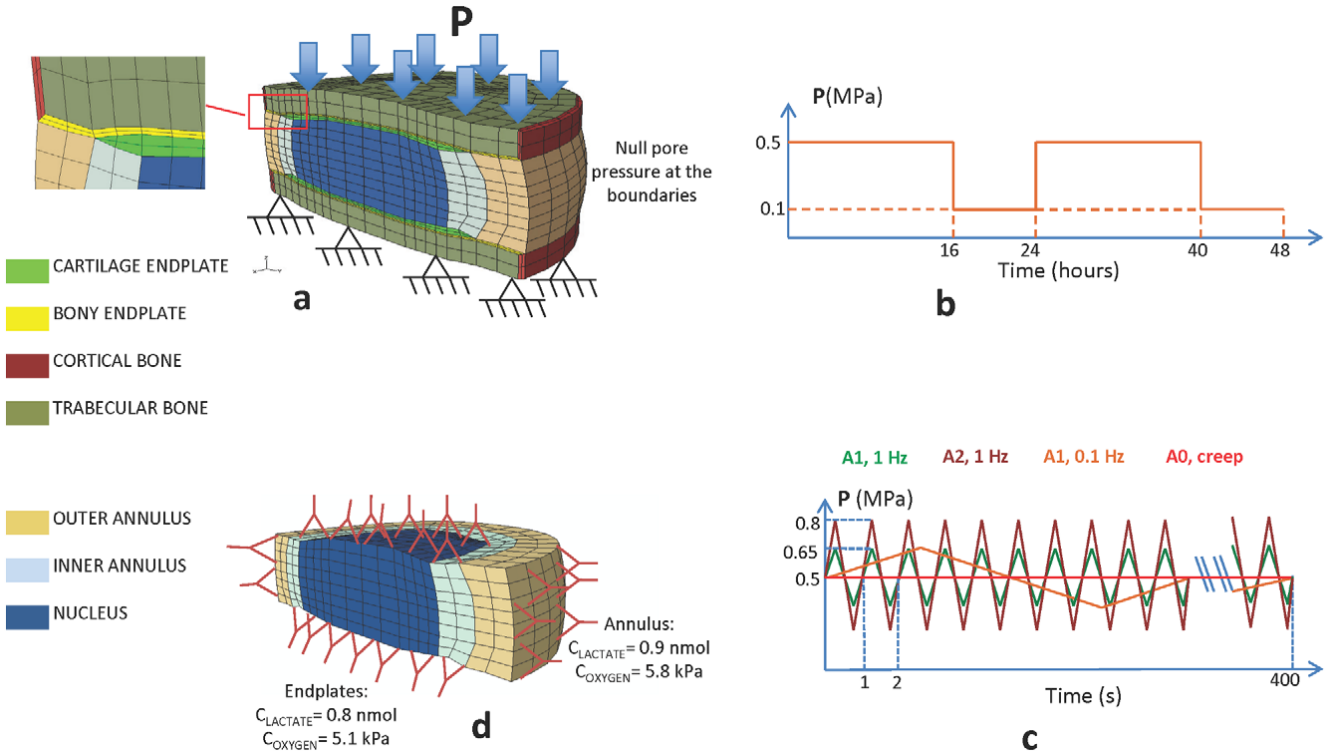
FE models, boundary conditions and loading modes used in the present study. (a) Poromechanical FE model for the IVD with all the subtissues modeled and boundary conditions applied for all the simulations; (b) load history for the diurnal cycle simulation; (c) load history for the cyclic frequency and amplitude comparison; (d) FE transport model with the applied boundary conditions. The red dots indicate the node were the results were calculated.

The model formulation (see Methods) allowed us to study the relevance of mechanical and metabolic couplings with different loading modes, under healthy properties and under DDCs. A sketch of the boundary conditions applied to the poromechanical and transport FE models are visible in Fig. 1a and 1d.

### Interaction between mechanics and metabolic oxygen transport in the healthy disc

Poromechanical and transport properties corresponding to a “healthy” disc were used (Table 1). The compressive creep behavior of the healthy IVD model in terms of vertical height change and nucleus pulposus (NP) pore pressure were validated in a previous study [22] against published experimental results [23]. To explore the mechano-transport responses of the models, a simple diurnal cycle consisting in 16 hours of creep under compression at 0.5 MPa and 8 hours of rest at 0.1 MPa of compressive stress was applied and repeated during two days (Fig. 1b).

**Table 1:** Set of poromechanical and transport properties for the simulated healthy disc and degenerated disc.

	ϕ_0_	k_0_ [mm^4^ N^−1^ s^−1^]	M	L	G [MPa]	K [MPa]	Δπ [MPa]	ρ_cell_ [10^6^cells mm^−3^]
Healthy IVD properties (▴H = 13.7 mm)								
Outer AF	0.73*	0.0002◊	1.18◊	-	0.28**	0.37**	-	0.063#
Inner AF	0.78*	0.0002◊	1.18◊	-	0.28**	0.37**	-	0.048#
NP	0.83*	0.0009‡	8.5++	-	0.12**	0.16**	0.15♠	0.032#
Degenerated IVD properties (▴H = 12.3 mm)								
Outer AF	0.57*	0.0002◊	1.18◊	-	0.41**	0.55**	-	0.048#
Inner AF	0.6*	0.0002◊	1.18◊	-	0.41**	0.55**	-	0.032#
NP	0.71*	0.0009‡	8.5++	-	0.19**	0.25**	0.05♠	0.02#
Bone and endplates properties								
CEP	0.8+	0.0025+	4.63+	0.08+	7.14‡	33.3‡	-	-
BEP	0.05++	26800$	-	-	3846++	8333++	-	-
Cortical	0.05++	5$	-	-	3846++	8333++	-	-
Trabecular	0.8++	26800$	-	-	42++	56++	-	-

*: Porosity based on an interpretation [57] of experimental results of [26]–[29] for healthy and degenerated IVDs.

# Cell densities are homogeneous in each subtissue. Data from [58], corrected by a living cell rate of 80% in the case of a healthy disc and 40% in the case of a degenerated disc [24].

**+:** evaluation from [35] based on experimental cartilage results [59].

**: from [60] for the healthy case and from [1], [30] for the degenerated one.

**‡:** from [30] for healthy NP case (values were not altered with degeneration by assuming the same AF permeability behavior [1]).

**++:** assumed in [17].

**♠:** Based on [61].

**▴:** The height H of the degenerated disc model was reduced by 10% to that of the healthy disc model based on an average 5% of disc height reduction for each grade of degeneration [38]. A moderate degeneration case, c.a. grade 3–4, was therefore simulated.

**‡:** Values were taken from [33].

$ Taken from [54], [62].

**◊:** from [1].

To establish the role of mechanics in transport predictions during a diurnal cycle, the healthy disc was tested with and without the abovementioned loading. When mechanical deformation was not considered, diffusivities were constant during the simulation and calculated from the initial porosities reported in Table 1 (Eq. (8) in Methods). With mechanical loading, the roles of strain-dependent diffusivity, changes in diffusion distances and advective transport were also computed. The effect of metabolic coupling with lactate was assessed for the healthy disc by comparing with a simulation where pH was constant and equal to 7.1, where oxygen reaction depends only on oxygen availability (see Eq. (10) in Methods).

**Figure 2:**
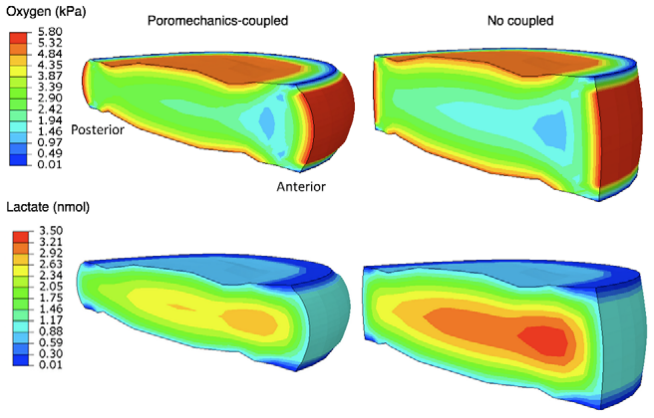
Distributions of the interdependent oxygen and lactate concentrations. Distributions are computed at the end of the 16-hours creep, with (left) and without (right) poromechanical coupling. With poromechanical coupling (left) both oxygen and lactate transport equations were solved over time taking into account the current deformed geometry.

**Figure 3:**
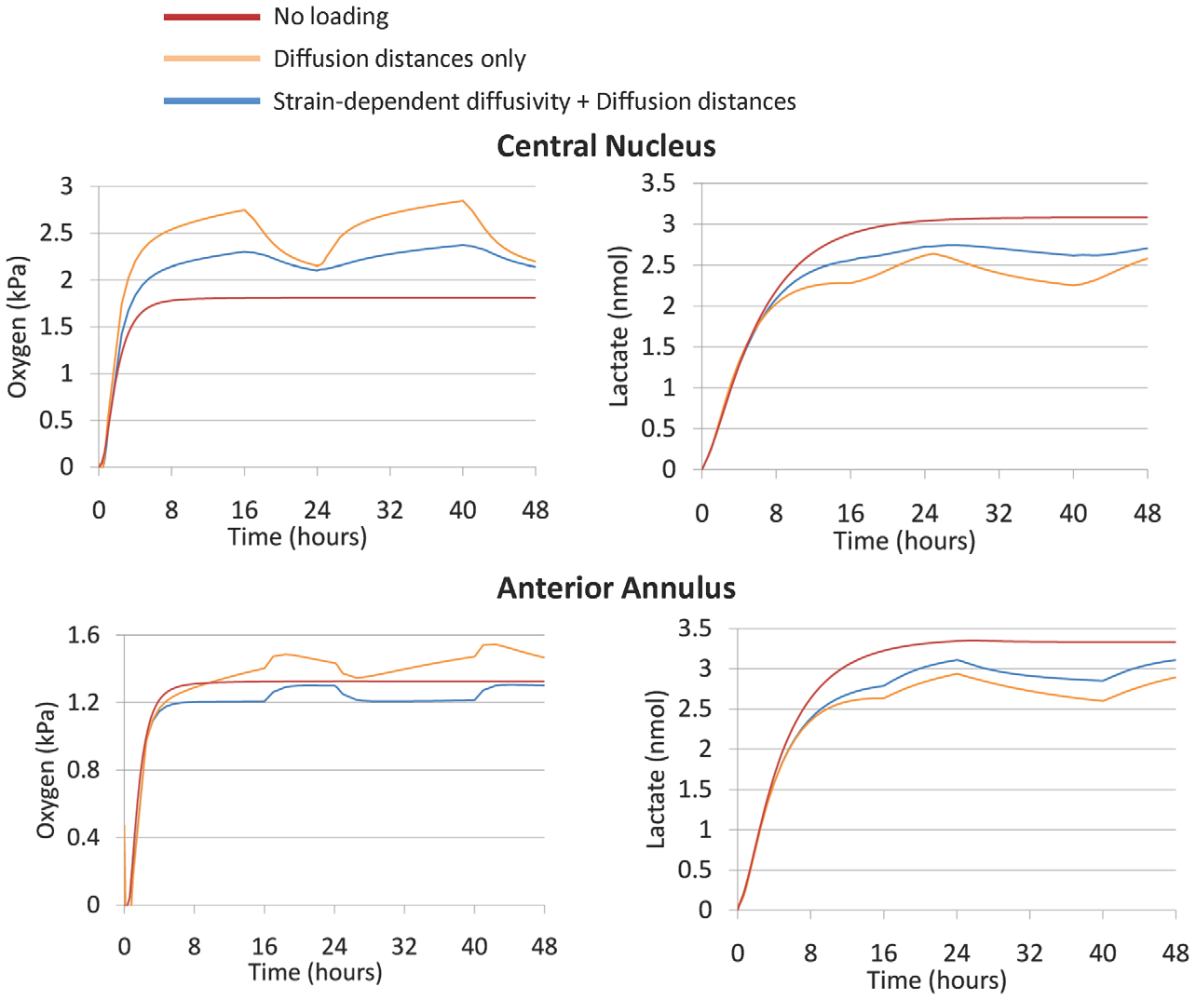
Effect of strain-dependent diffusivity and diffusion distances on oxygen and lactate. Comparisons in terms of oxygen and lactate concentration in the AF and NP of the healthy disc model under three cases: no loading, loading with a reduced disc height, and loading with reduced disc height and strain-dependent diffusivity under the diurnal cycle loading mode for two days simulated.

**Figure 4:**
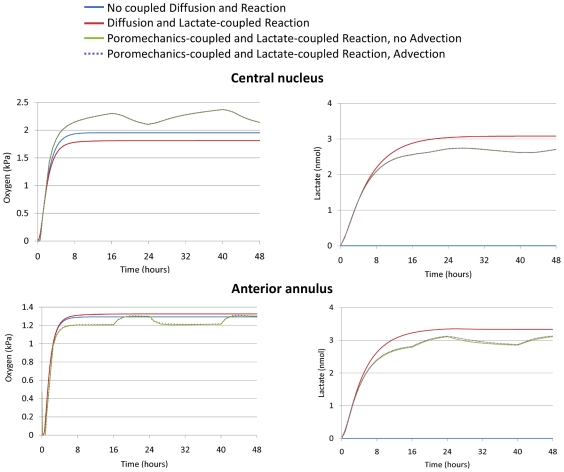
Effect of different couplings on oxygen and lactate. Comparisons in terms of oxygen and lactate concentration in the AF and NP of the different combinations studied in terms of couplings under the diurnal cycle loading mode for two days simulated.

Fig. 2 shows oxygen and lactate distributions in a sagittal section of the disc model with and without poromechanics-transport coupling, and for a transient analysis corresponding to the end of the second 16-hour creep period. For all cases, the anterior annulus fibrosus (AF) presented the lowest oxygen concentrations and the highest lactate levels. Local oxygen concentration is shown over time (Fig. 3 and 4) for two nodes at the central NP and the anterior AF (red dots in Fig. 1d). With initial conditions of zero oxygen and lactate within the disc, steady state concentrations were reached in approximately 16–18 hours without mechanical loading. When mechanical deformation was considered, a steady-state solution never occurred. Instead, a repetitive pattern was identified following loading and recovery phases. In all cases, with mechanical coupling, maximum oxygen and lactate concentration changes occurred at the end of the creep compression. Shortening the diffusion distance by 10% in the undeformed healthy disc height with constant diffusivities increased the oxygen levels in both the central NP (up to 57%) and the anterior AF (up to 11%). Simultaneously, the lactate concentration decreased by a maximum of 27% in the NP and 22% in the AF, when compared to the undeformed case (Fig. 3). Including strain-dependent diffusivities resulted in a subsequent oxygen decrease in both the NP (up to 17%) and the AF (up to 18%) with a coupled counter-balanced lactate increase (up to 16% in the NP and 10% in the AF), in comparison to the model with decreased diffusion distances and constant diffusivities. Merging together both strain-dependent diffusivity and diffusion distance changes caused an oscillating increase of oxygen for the central NP with a peak of 31% compared to the undeformed case, while a maximum of 9% oxygen decrease was calculated for the anterior AF. For lactate, with both strain-dependent diffusivities and distance changes, a similar decrease in both the anterior AF and the central NP up to 15% was found, compared to the undeformed case (Fig. 3). Advective effects were insignificant. Finally, when neglecting the lactate-dependence (via pH changes) of oxygen reaction, we found a 2.5% decrease in oxygen concentration in the anterior AF and 8% increase in the central NP, with respect to the lactate-coupled solution without poromechanical coupling (Fig. 4).

### Study of cyclic compression effect on metabolic oxygen transport in the healthy disc

A cyclic compression with an average load of 0.5 MPa was applied during 400 seconds. Two different frequencies (1 Hz and 0.1 Hz), and two different amplitudes (A1 and A2) at 1 Hz were considered (Fig. 1c). The initial conditions of the transport model during the dynamic loading simulations corresponded to the preconditioned solution of lactate and oxygen at the end of the two days of diurnal cycles. The different dynamic loading modes were compared to each other and with a creep compressive loading of 400 seconds (A0 in Fig. 1c). We computed negligible effects (<0.5% relative differences) of amplitude A1 vs. A2, frequency 1 Hz vs. 0.1 Hz, and amplitude A1 and A2 vs. creep loading amplitude A0 (data not shown).

### Sensitivity of the transport model to mechanical and metabolic parameters related to DDCs

The parameters shown in Table 2 were varied one by one in order to compare oxygen and lactate profiles to a base model (with the healthy properties in Table 1), including all mechanical and metabolic couplings. Such changes were related to DDCs reported in experiments to assess the sensitivity of the model under realistic parameter ranges. A reduction in the overall IVD cell density (both AF and NP) from the base model was simulated based on the experimental observation of substantial increase in cell apoptosis [24] and decrease in cell activity [25] with degeneration. Porosity decrease in both AF and NP from healthy to degenerated was also reported [26]–[29]. Values were thus reduced to simulate degeneration and study the sensitivity of the model within this range. Similarly, since solid phase stiffening during degeneration was reported in both AF [1], [27] and NP [26], the sensitivity of the model predictions was evaluated to global solid phase stiffness increase in both sub-tissues. Finally, a decrease in pH from healthy to degenerated IVDs and a decrease in NP swelling pressure [30] (related to the proteoglycans loss [25]) were studied as detailed in Table 1 and 2. All the above parameter changes, following the corresponding experimental sources, referred to a grade of degeneration larger than 2.5 in commonly used grading systems [31], [32].

**Table 2:** Set of poromechanical and transport properties for the sensitivity study.

	POROSITY DECREASE	CELL DENSITY DECREASE [10^6^ cells mm^−3^]	pH DECREASE	CEP PERMEABILITY INCREASE *k_0_* [mm^4^ N^−1^ s^−1^]	STIFFENING OF AF AND NP SOLID PHASE [MPa]	NP SWELLING PRESSURE DECREASE [MPa]	CEP STIFFNESS DECREASE [MPa]	BEP STIFFNESS DECREASE [MPa]
Base Model								
AFO	0.73	0.063	7.1**	-	*G* = 0.28; *K* = 0.37	-	-	-
AFI	0.78	0.048	7.1**	-	*G* = 0.28; *K* = 0.37	-	-	-
NP	0.83	0.032	7.1**	-	*G* = 0.12; *K* = 0.16	0.15	-	-
CEP	-	-	-	0.0025	-	-	*G* = 7.14; *K* = 33.3	-
BEP	-	-	-	-	-	-	-	*G* = 3846; *K* = 8333
Altered Model								
AFO	0.57	0.048	6.2**	-	*G* = 0.41; *K* = 0.55	-	-	-
AFI	0.6	0.032	6.2**	-	*G* = 0.41; *K* = 0.55	-	-	-
NP	0.71	0.02	6.2**	-	*G* = 0.19; *K* = 0.25	0.05	-	-
CEP	-	-	-	0.025▴	-	-	*G* = 2.14‡; *K* = 2.53‡	-
BEP	-	-	-	-	-	-	-	*G* = 384.6†; *K* = 833.3†

For each property except CEP and BEP stiffness, the values were chosen at the limits of the range of degeneration grades reported in the literature. The “healthy disc” model is always identified as the base model and the “degenerated disc” model as the “altered” one. For all other properties not listed in this table, refer to the “healthy disc” properties in Table 1. AFI  =  inner annulus fibrosus, AFO  =  outer annulus fibrosus, NP  =  nucleus pulposus, CEP  =  cartilage endplate, BEP  =  bony endplate.

For all other properties not listed in this table, refer to the “healthy disc” properties in Table 1, except:

**▴:** Altered value increased of one order of magnitude with degeneration (assumed based on [25]).

**: Values from [61].

**‡:** altered values assumed in [16].

**†:** altered values assumed in [34].

AFI = inner annulus fibrosus, AFO = outer annulus fibrosus, NP = nucleus pulposus, CEP = cartilage endplate, BEP = bony endplate.

The sensitivity of the metabolic transport outcomes was also evaluated with parameters from other IVD computational models [16], [33]–[35], even though such values might lack experimental support: (i) cartilage endplate permeability (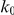, see Methods), varied over several orders of magnitude in the literature [36], [37], [25] and thus was increased one order of magnitude (as reported during ageing and degeneration [25]); (ii) cartilage endplate stiffness and Poisson's ratio values taken from [33] were both lowered up to the values reported in [16] and (iii) bony endplate (BEP) stiffness, taken in the base model as 10 GPa [16] was decreased one order of magnitude as in [34].

**Figure 5:**
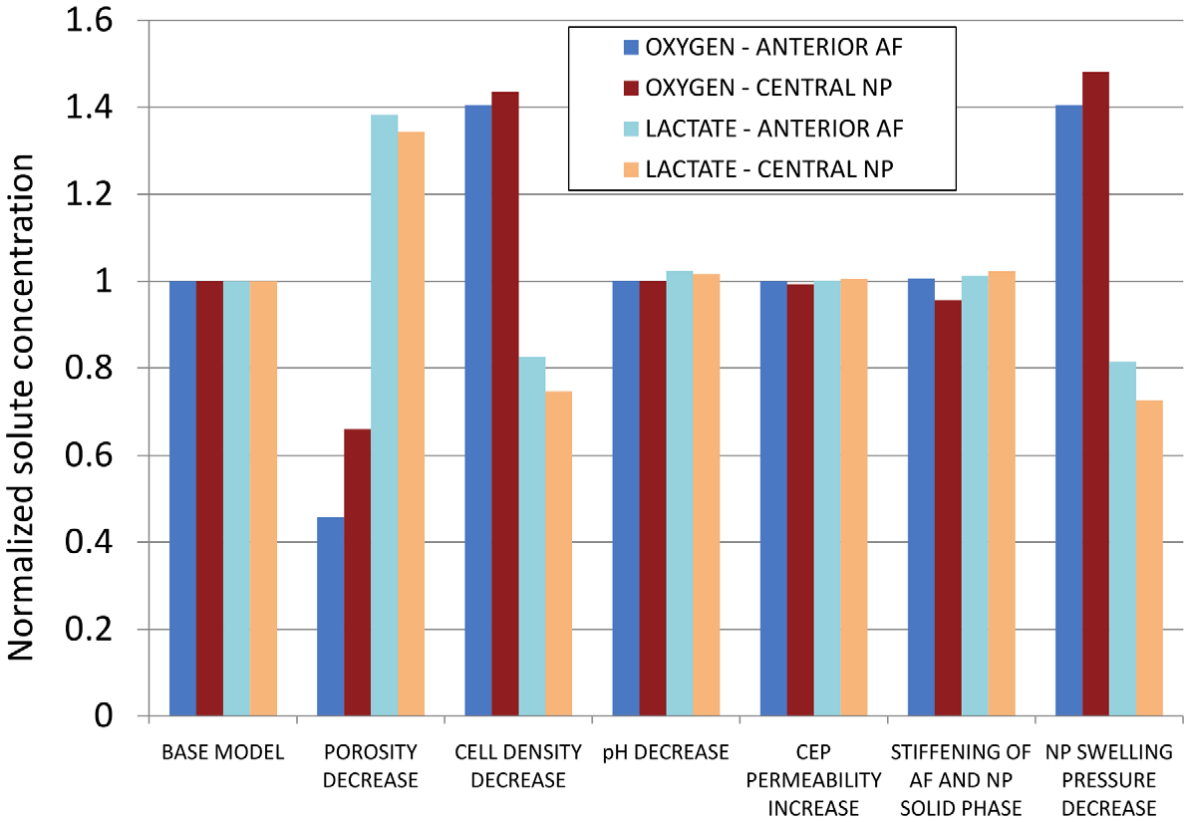
Results of the sensitivity study. Oxygen and lactate concentrations in the AF and NP are normalized to the base model.

Fig. 5 shows the sensitivity in terms of solute (oxygen or lactate) concentration under a given parameter change, normalized to the base model value, for both AF and NP. Fig. 5 refers to results at the end of the two simulated days. Within both the AF and the NP, oxygen and lactate concentrations were significantly more sensitive to porosity, cell density and swelling pressure changes than to variation of the other parameters, i.e. CEP permeability, pH and solid phase stiffening. In average, over both the AF and the NP, the oxygen concentration decreased by 44% and the lactate concentration increased by 36% when porosity was reduced. When cell density was reduced, the oxygen relatively increased by 42% and the lactate decreased by 21%. When the NP swelling pressure was reduced, a 44% relative increase in oxygen and a 23% relative decrease in lactate concentration were computed. As for the remaining parameters, less than 3% of relative changes in solute concentrations were found. Finally, CEP and BEP stiffness variations gave differences in oxygen and lactate concentrations lower than 0.2% (data not reported in Fig. 5). The sensitivity results in Fig. 5 were similar to those found at the end of the sustained creep (data not shown).

### Simulation of multiple disc degenerative changes

**Figure 6:**
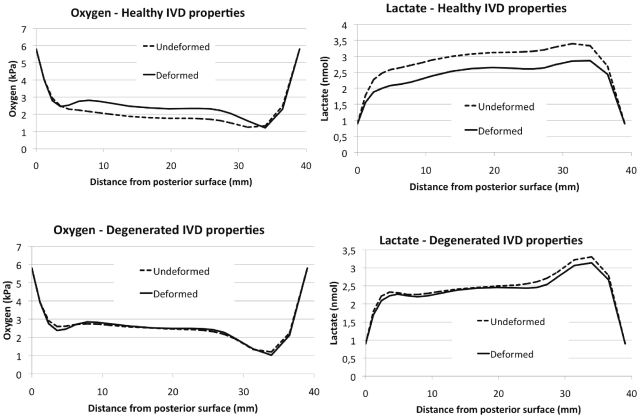
Effect of healthy and degenerated disc properties. Oxygen and lactate levels for the IVD mid-height and as a function of the anterior-posterior position for simulated healthy (top) and degenerated (bottom) disc properties.

The healthy base model was compared with a model in which all the relevant DDC-related material properties reported in Table 1 were considered, i.e. disc height, porosity, cell density and swelling pressure reduction, and the solid phase stiffening (Table 1). However, the histological distinction between the two AF and NP subtissues was preserved, assuming a mild or moderate degenerated disc with a grade of 2.5–3 in the Thompson scale [38], [32]. Fig. 6 shows the oxygen and lactate concentrations predicted at the end of the second 16-hours creep compression along the mid-transversal anteroposterior paths of both the healthy and degenerated disc models. Transport equations and mechanical deformations were alternatively coupled (deformed) and decoupled (undeformed). While mechanical coupling tended to favor oxygen concentration and limit lactate accumulation within the healthy disc, it had only scarce effects within the degenerated IVD model.

## Discussion

A computational approach based on poromechanics and metabolism was developed and applied to investigate oxygen transport within the human IVD. First, the relevance of the mechanical and metabolic couplings was evaluated. Second, different degenerative changes were simulated with the proposed method to assess their effects on metabolic oxygen transport.

In the healthy disc, predictions for oxygen distribution were most sensitive to mechanical coupling, this effect being predominant over that of the metabolic coupling. Advective transport by fluid movements was negligible in daily loading modes and after short period of cyclic compression. Mechanical effect acted via diffusion by local porosity changes, affecting diffusivities, and by global changes in geometry, affecting the diffusion distances. Unsurprisingly, mechanical effect was thus mainly observed when large volume changes were present, which was favored by healthy disc material properties. Disc height, cell density, NP swelling pressure and porosity DDCs affected more oxygen transport than AF and NP solid-phase stiffening.

Effects of (i) daily disc deformation, and (ii) disc height change due to fluid loss with aging and degeneration on transport were studied. In the central disc, we found a general increase in oxygen concentration, induced by both sustained compression and permanent geometrical change. Such phenomena occurred in concomitance with a decrease of lactate levels, as a consequence of the oxygen-dependent lactate metabolic rate. These results are in agreement with other predictions [21] in which oxygen enhancement and lactate reduction were found following a volume loss, simulated by altering the IVD model dimensions. Our study further focused on the dual effect of a permanently reduced height together with daily deformations due to loading, as happens in a degenerated disc under sustained compression. While volume changes in the simulated healthy IVD during the load-recovery phases had a remarkable effect on solute levels, in the degenerated disc model, such effect was reduced because of decreased fluid content and swelling pressure, as well as increased influence of the solid phase (Fig. 6). This outcome could indicate how compression, here related to the fluctuation of solute concentrations, could be seen as a “healthy” condition for the disc [7]. Interestingly, the beneficial effect of compression through volumetric deformations could partly explain the fact that no differences in cell density were observed between male and female discs [3]: despite the increased diffusion distances in male discs, if the disc is sufficiently healthy, large dimensions would allow for increased deformability and improved disc maintenance through mechano-transport coupling. Moreover, the observed reduction in the normal load-dependent solutes pattern that occurred when degenerated material properties were simulated could be seen as an IVD degenerative catalyst.

**Figure 7:**
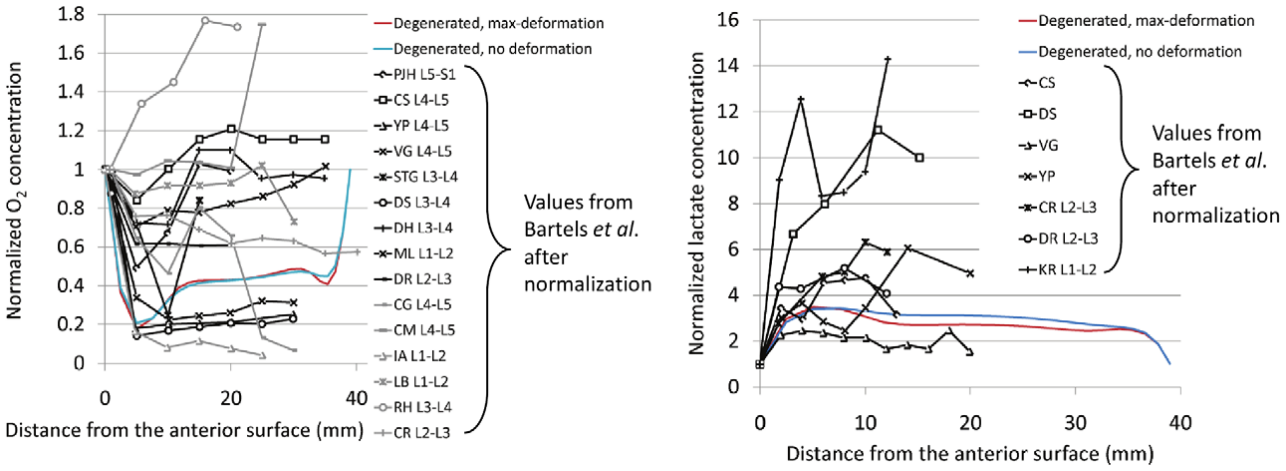
Comparison with oxygen and lactate measurements from literature. Oxygen and lactate normalized concentration from published experimental data on human patient with back pain and scoliosis [13] (patient designation duplicated from [13] followed by the level of the IVD where concentrations were measured) compared with the model results from the present study (case with all DDC simulated, “maximum deformation” refers to the end of the second sustained compression period and no deformation to the steady state solution).

Distributions of oxygen and lactate predicted by our 3D model are difficult to validate as it would require invasive and complicated experimental procedures. Nevertheless, minimum oxygen pressure at disc mid-height was about 1 kPa in all models, which was close to the minimum value found *in-vivo* [13] in patients with scoliotic and back pain (0.71 kPa). Also, the maximum lactate concentrations, predicted nearby 3.5 nmol, were within the measured experimental range of 2–6 nmol [13], and the location of minimum oxygen and maximum lactate contents were in agreement with other studies [17]–[19]. Fig. 7 shows the results of oxygen and lactate in the present study against the experimental range normalized with the boundary value found for each patient [13]. Normalization was performed because the solute concentrations reached within the disc strongly depend on the patient-specific vertebral blood supply. Although the comparison was made only with the lumbar discs, the experimental variability is still very high. However, a similar anteroposterior trend can be found between our study and the experimental results for both lactate and oxygen concentrations (in particular for experimental curves in black in Fig. 7). Such a comparison strongly suggests that the vertebral blood supply condition is a relevant factor that drives the absolute values predicted by any metabolic transport model and could also be relevant for the onset of possible degenerative changes [25]. Because of the large amount of calculations already performed in the present study, and because of scarce information in the literature about IVD boundary conditions in terms of oxygen concentration, such parameter was not explored. However, the sensitivity study of this work provides new information on the influence of parameter changes as measured in disc degeneration (stiffening of solid phase as well as disc height, fluid content, swelling pressure and cell density decreases).

The different responses of the AF and NP to mechanical coupling were related to the combination of subtissue-specific deformation modes and changes in diffusivity. In fact, the changes in diffusion distance caused an increase of oxygen and a decrease of lactate in both subtissues. Also, the strain-dependent diffusivity caused a decrease of oxygen and an increase of lactate when compared to a deforming disc with constant diffusivities, both in the AF and the NP. When strain-dependent diffusivity and shortening of diffusion distance were combined together, changes in solute concentrations were regionally opposite in the two substissues, because AF was significantly less sensitive than the NP to shortening of diffusion distance (blue lines vs. red lines in Fig. 3). In the anterior AF where the oxygen availability was already low, changes to diffusion-related parameters resulted in an oxygen deprivation. The location of such critical regions depends strongly on the disc geometry, distance from blood vessels, and regional water content loss, i.e. on patient specific characteristics. Finally, diffusion distance shortening due to mechanical loads, in the healthy IVD model gave 15% of disc height change after 7 hours under 0.5 MPa compression, which was comparable to *in vivo* deformations of 10–15% measured with comparable loads [39].

Fluid velocity enhancement for nutrients was negligible under higher frequency and amplitude loading modes. The highest fluid velocities were around 0.5 µm/s, at 1 Hz frequency and for the A2 load amplitude. Even if a particle was submitted to such velocity magnitude permanently during the simulated 400 seconds, the advective transport distance would have been 0.2 mm. Therefore, within the AF and the NP, considering both the low tissue permeability and the characteristic dimensions two orders of magnitude higher than the advective distance, fluid velocities are unlikely to transport small solutes. Moreover, AF and NP permeability changes (although not simulated as a DDC following the experimental findings of [1]) are known to have a negligible role in the displacement field calculation [40] and thus on the IVD volume changes. Therefore, the solute transport sensitivity is expected to be negligible via load-dependent diffusion. The negligible role of fluid velocity advection is congruent with other computational [16] and experimental evidences [11], [41], [12]. Yet, Huang and coworkers [17] predicted small solute enhancements due to dynamic loadings after 200 cycles and at a frequency of 0.1 Hz. However, advection and diffusion were not explicitly presented in the study of Huang *et al.* and dynamic vs. static loading differences could be also attributed in part to the loading-dependent diffusion. The endplates solute permeability change may also have played a role, while we did not simulate any alteration of solute input values at the endplates to avoid biasing the effect of the considered DDCs.

Recent computational studies on IVD metabolic transport have included glucose [18]–[21], which has a high influence on cell viability [20], [42]. Since we did not consider local cell matrix synthesis and viability, glucose was ignored. In this first approach we demonstrated that cell density disturbance had a significant effect, since it is related with both reactive (metabolic) terms for oxygen and lactate. Cell density decrease has also been related to poor nutrient supply due to abnormalities of the CEP [3]. Thus, glucose concentration may play an important role if coupled to cell death. Likewise, high lactate concentrations resulting from a poor waste product removal could be detrimental to cell survival and should be taken into account via a pH and/or glucose dependent cell death, which would lead to the understanding of the degenerative processes under a cell biology-based chronology. On the other hand, since the total number of cells have also been shown to increase with degeneration [25] our simulated decrease in cell density could be optimistic with respect to a situation in which a non-functional cell number increase would occur and thus, a more severe condition in terms of oxygen consumption.

In summary, the developed method to investigate the poromechanics and metabolic-coupled oxygen transport revealed that mechanical loads can significantly affect oxygen and lactate predictions when large and prolonged volume changes are involved. Such phenomenon was caused by the deformation-dependent nature of both tissue diffusivity and diffusion distances. The mechanism was regional- and solute-dependent within the disc. By applying the proposed approach together with IVD degenerative changes, we conclude that cell density drop, disc height reduction and decrease in NP swelling pressure due to loss of proteoglycans would significantly alter the interactions between mechanical loading and disc nutrition and be detrimental to the diffusion of nutrients. Thus, disc nutrition is most likely part of the synergy suggested between disc degeneration and physical activity [8], [2]. In a healthy disc, it was found that mean compressive load variations in a daily cycle could be beneficial to disc maintenance. The approach could be used in regenerative and preventive medicine as a patient-specific numerical tool to explore the chronology of events in disc degeneration, in which the input parameters (such as geometry, diffusivity, hydration) could be derived from diagnostic images. The computational framework developed could also serve to study cell-loaded disc substitute materials.

## Methods

A 3D FE poromechanical model coupled with an ADR transport model was created. The geometry of a L4–L5 IVD model was taken from an accurate spinal segment FE model [43]. All the relevant subtissues were represented: nucleus pulposus, annulus fibrosus, cartilage endplate (CEP), bony endplate (BEP), cortical bone and trabecular bone. For both the AF and NP, the solid porous skeleton was treated as a compressible Neo-Hookean material. In such a poro-hyperelastic formulation [44], the solid grains were intrinsically incompressible and the porous material was considered to be saturated with an incompressible fluid phase, i.e. water. The total stress tensor 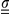 caused by external loadings was the superimposition of the porous solid stress and the fluid pore pressure, *p*, that were respectively derived from a strain energy density function, 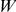
_,_ and Darcy's law:
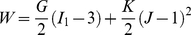
(1)

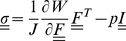
(2)

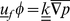
(3)In the above equations, *G* and *K* are respectively the shear and the bulk modulus, 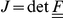 with 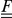 the deformation gradient tensor, 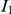 is the first strain invariant, 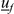 is the pore fluid velocity, 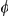 is the tissue porosity, and 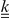 is the hydraulic permeability tensor of the tissue. With both phases being incompressible, in a large strain formulation the porosity varies with deformation with respect to an initial value 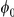 as 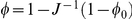. Soft tissues permeability is highly strain-dependent and different exponential constitutive laws have been proposed [45], [46] relating the isotropic hydraulic permeability with the porosity, the volumetric strain 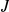 and with an initial value 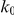. The following laws were used:
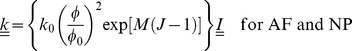
(4)

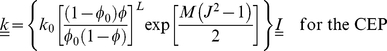
(5)being 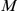 and 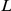 empirical coefficients, and 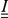 the second-order unit tensor.

In the AF, the porous solid strain energy density, 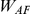, was the sum of Eq. 1, and an additional term accounting for the anisotropic and nonlinear fibre-induced strengthening [47]:
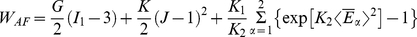
(6)The additional term depended on fibre stiffness parameters 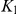 and 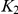 and on the tension-only quantity 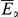, active only in two opposite fibre directions (*α = 1,2*) and related to the fibre criss-cross distribution observed in AF. Different distributions of fibre orientation explained the regional differences in AF mechanical behaviour as detailed in our previous study [22].

For the NP, proteoglycan-induced NP swelling was described by considering the fluid pressure as a sum of the water chemical potential, 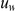, and a swelling-pressure related term, 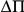
_,_ that was assumed constant during deformation [48]:
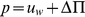
(7)For the ADR transport model, the tissue-averaged continuity equation can be expressed in the following fashion [49]:
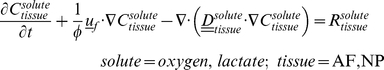
(8)where 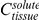 is the volume-averaged solute concentration, 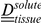 is the tissue diffusivity, and 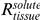 is the metabolic reactive term.

Solute transport was solved by using a thermal-transport analogy [50]. To take into account advection and volumetric changes, a sequential approach was implemented so that the poromechanical results affected the transport solution. A Mackie-Meares diffusivity 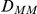 was used [51], [52], which relates the volume-averaged isotropic diffusivity of each solute with the updated porosity of the medium and the solute diffusivity in water 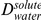:
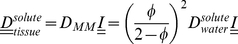
(9)Fluid velocities were computed from the poromechanical analysis and used as input for the advective term. All calculations were performed with ABAQUS 6.9 (Simulia, Providence, RI, USA). However, since this commercial package did not allow modeling advection together with strain-dependent diffusion, the advective-diffusive flux was modified via user subroutine by adding a stabilization term to avoid oscillations in the results [53]:
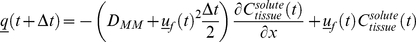
(10)This approach, dependent on the temporal and spatial discretizations, was verified against theoretical results using quadratic elements that fulfilled the condition 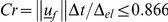, where 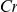 was the Courant number, and 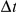 and 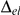 the time step and the mean element length, respectively.

**Figure 8:**
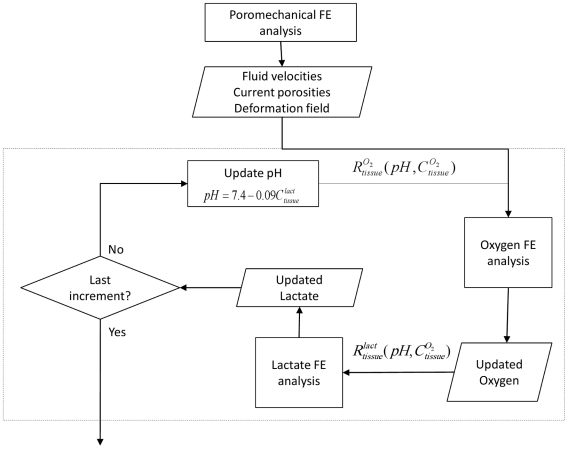
Poromechanics-transport coupling scheme. Sequential coupling scheme between poromechanical FE model and transport FE model (dashed line box). The latter considered both oxygen and lactate FE analysis coupled to account for IVD metabolic reactions.

Finally, metabolic reactions from experiments on IVD cells [10] were used for:

- pH- and Oxygen-dependent oxygen cell consumptions
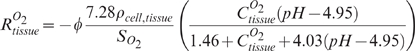
(11)
- lactate production
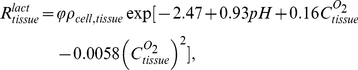
(12)where 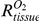 is in *kPa/h*, 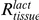 is in *nmol/h*. 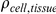 is the cell density for the considered tissue, 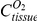 is the oxygen concentration in *kPa*. The pH was coupled to the lactate concentration, 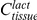, expressed in *nmol* via the equation [21]:
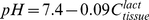
(13)Cortical and trabecular bone properties (considered only in the poromechanical model) are based on [54], [16] and are representative of the properties measured in bone tissue experiments [55], [56]. Thus, such properties were not altered since they are expected not to influence the transport results and the study of their influence is out of the scope of the present study. On the contrary, bone and cartilage endplate properties were studied in a sensitivity fashion (see Table 2). Shear and bulk moduli for soft tissues were calculated from the zero-strain compressive modulus 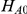 as 
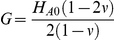
 and 
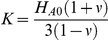
 [27], [30] by assuming a Poisson's ratio 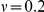 [30] in all cases. The boundary conditions for the transport equation were taken from literature [21], being 5.8 kPa oxygen partial pressure and 0.9 nmol lactate concentration at the external AF edges and 5.1 kPa and 0.8 nmol at the cartilage endplate boundaries corresponding with the cartilage endplate. Such concentrations were not altered with degeneration. The poromechanics-transport-metabolism sequential coupling is illustrated schematically in Fig. 8. As indicated, the transport routine starts with the oxygen FE simulation. At each increment, the resulting oxygen concentration is used to update the lactate production and the following FE lactate results are used in turn to update pH levels and, consequently, oxygen consumption at the next increment. We verified that changing the routine order (i.e. lactate and oxygen FE analyses order) did not affect the results for the chosen time increments (data not shown).
